# Growth Performance and Carcass Traits of Cobb 500 Broiler Chickens Fed With Different Levels of Cinnamon (*Cinnamomum cassia*) and Fennel (*Foeniculum vulgare* Mill.) Powders as Natural Feed Additives

**DOI:** 10.1155/sci5/9456440

**Published:** 2026-04-24

**Authors:** Abebe Bezahegn, Berhan Tamir, Gezahegne Mamo

**Affiliations:** ^1^ Department of Animal Sciences, College of Agriculture, Oda Bultum University, Chiro, Ethiopia, obu.edu.et; ^2^ Department of Animal Production Studies, College of Veterinary Medicine and Agriculture, Addis Ababa University, Bishoftu, Ethiopia, aau.edu.et; ^3^ Department of Veterinary Microbiology, Immunology and Public Health, College of Veterinary Medicine and Agriculture, Addis Ababa University, Bishoftu, Ethiopia, aau.edu.et

**Keywords:** broiler chickens, carcass traits, cinnamon, fennel, growth performance

## Abstract

The natural and organic poultry production industry is growing. This is in response to rising consumer demand for poultry products that are natural and organic. The purpose of this study was to examine the effects of dietary inclusion of cinnamon and fennel powder as natural feed additives on growth performance and carcass traits of broilers. A total of 210 day‐old chicks were randomly assigned to five dietary treatments. The control group (T1) was provided with a standard diet containing no fennel seed powder (FNSP) and cinnamon bark powder (CNBP), while the other groups received a standard diet plus 0.75% FNSP (T2), standard diet plus 1.75% FNSP (T3), standard diet plus 0.75% CNBP (T4), and standard diet plus 1.75% CNBP (T5). Feed intake data were taken daily, while the body weight change was measured at a weekly interval. At the end of the experiment, three chickens from each treatment were picked and slaughtered for carcass traits measurements. The results revealed that broilers fed a diet containing 1.75% CNBP achieved significantly (*p* = 0.036) higher final body weight gain of 3243 ± 58.88 g, average daily weight gain of 70.88 ± 1.20 g and the best feed conversion ratio (*p* = 0.033) of 1.44 ± 0.03 at the end of the experiment when compared to the control group. Additionally, significantly higher dressed (*p* = 0.039) and eviscerated (*p* = 0.008) weights were found in 1.75% CNBP compared to the control. Furthermore, broilers in CNBP at 1.75% and 0.75% had significantly higher (*p* = 0.001) breast, thigh, and whole leg weight over the control and other treatments, except for the 1.75% FNSP group, which had significantly higher breast weight than the control. In general, incorporation of cinnamon powder in broilers’ diet, particularly at a level of 1.75%, showed significant improvement in overall growth performance and carcass traits.

## 1. Introduction

Poultry industry is one of the fastest growing sectors, especially in low‐ and middle‐income countries. In rapidly intensifying poultry farms, particularly in developing countries, producers often use antibiotics without veterinary consultation to enhance growth and maximize economic gains [1]. However, the indiscriminate use of antibiotics driven by economic interests led to the misuse of antibiotics, including continuous use during the withdrawal period, beyond the application range and superimposed antibiotics incorrectly [2]. In addition, there is conclusive evidence suggesting that the expanded usage of antibiotics in animal feed is associated with a range of issues, including drug residues and bacterial resistance, which pose significant threats to the long‐term development of animal husbandry and human health [2, 3]. Besides, consumers are seeking not only safe or nutritious food products, but they also demand natural, organic, and functional products [4]. Thus, various studies have focused on the use of alternatives to antibiotics, such as phytogenic (plant origin) compounds, as potential natural growth‐enhancing additives that offer several advantages in poultry nutrition [5]. Among these plant origin feed additives, fennel (*Foeniculum vulgare* Mill.) and cinnamon (*Cinnamomum cassia*) are considered as natural growth promoters in the poultry farming industry with desirable attributes without any adverse effects. Several studies have explored natural compounds such as fennel as alternatives to synthetic antibiotics in broiler feeds [6–8] and cinnamon [9–11], all aimed at enhancing performances in terms of growth, feed efficiency, and carcass yield.

Fennel seeds and cinnamon barks are aromatic plant‐derived products that have recently gained consideration as a beneficial natural feed additive in livestock and poultry nutrition in addition to their uses as a flavor in cooking all around the globe. Fennel seeds have a variety of biological effects in broilers, including improved performance due to the presence of active ingredients such as anethole, estragole, limonene, and fenchone [12]. Cinnamon is a source of bioactive components, including cinnamaldehyde, eugenol, and cinnamyl acetate, among numerous other valuable bioactive phenolic compounds [11, 13]. Based on the study results reported by Meligy et al. [14] in broilers and Bakeer et al. [15] in fattening rabbits, the use of such phenolic compounds as a natural feed additive exhibits a wide range of physiological properties, including antioxidant activities, enhancing nutrient digestibility by stimulating the activity of digestive enzymes, and contributing to improved growth and overall health.

Furthermore, Ali et al. [10] suggested that cinnamon inclusion in poultry feed as a natural feed additive improves growth performance, carcass traits, and overall health of broiler chickens. Similarly, Nassar et al. [8] reported that the addition of fennel to the diets of broilers improves body weight (BW) gains, feed‐to‐gain ratios, and weights for the carcass yield. Most of the previous studies investigating fennel and cinnamon have primarily focused on low inclusion levels. For instance, fennel has been investigated at rates ranging from 0.15% to 0.45%, which resulted in no improvement in broilers’ growth performance [16]. In addition, no significant difference was observed in all carcass characteristics except the true stomach and pancreas weight percentage in broilers fed fennel at 1, 2, and 3 g/kg of inclusion levels [17]. Similarly, cinnamon inclusion levels between 2 and 4 g/kg did not influence the feed conversion ratio (FCR) and carcass percentage except for BW gain [18]. Moreover, the inclusion of cinnamon at levels of 2–6 g/kg did not affect slaughter weight and carcass yield in broiler chickens [19].

On the other hand, some of the studies have explored higher inclusion levels. For instance, fennel seeds at 1.6% and 3.2% were used, which improved the growth performance under a heat stress condition, but no differences were noticed among the treatment groups under a normal temperature condition in broiler chickens [6]. Additionally, cinnamon at 2%–8% [20] and 1%–5% [21] showed no significant effects on growth performance and carcass traits of broilers. Due to the inconsistencies in the results reported depending on the inclusion rates in the previous studies, and to find the optimum inclusion level, the present study determined moderate inclusion levels of 0.75% and 1.75% for both cinnamon and fennel in the basic diet. Therefore, this study was designed to examine the effects of fennel seed powder (FNSP) and cinnamon bark powder (CNBP) additives on the feed intake, BW change, and carcass traits in broiler chickens.

## 2. Materials and Methods

### 2.1. Study Area

This study was conducted at the Poultry Research Unit of the College of Veterinary Medicine and Agriculture, Addis Ababa University, Bishoftu campus, Ethiopia. Bishoftu is situated at 47 km southeast of Addis Ababa at an altitude of 1900 m above sea level, latitude of 8°44′N and longitude of 38°57′E. The average annual rainfall is 851 mm with an average minimum and maximum temperature of 8.9°C and 28.3°C, respectively, and the average relative humidity is 58.6% [22]. The study was conducted for 49 days, including 5 days of adaptation period from May 23 to July 10, 2024.

### 2.2. Preparation of Experimental Feed Additives

Dried barks of cinnamon were purchased from Akaki market in Addis Ababa, whereas fennel seeds were purchased from Chiro market in West Hararghe, Oromia, Ethiopia. The dried cinnamon barks were crushed into small pieces with the aid of a hammer mill for an easy drying process. After complete drying at room temperature for 3 days, the fennel seeds and cinnamon bark were powdered using a multipurpose electronic grinder. Later, the powdered fennel and cinnamon were placed in a strictly airtight polythene bag and stored in a cool, dry place until incorporation in the broilers’ diet. The powdered fennel and cinnamon were added to the standard feed and mixed homogeneously to ensure uniform distribution.

### 2.3. Study Design

In this experiment, a total of 210 day‐old broiler chicks were randomly divided into five treatments (T1, T2, T3, T4, and T5) in a completely randomized design (42 chicks per treatment with three replications of 14 chicks per replicate). The chicks were raised on a commercial broiler starter diet for five consecutive days in order to encourage feed and water intake and to acclimatize the chickens to the new environment before commencement of the experimental trial. At the commencement of the experiment, the chickens in each replicate were weighed to determine their initial weight. Treatment (T1) designated as the control was provided with a standard diet containing no FNSP and no CNBP throughout the experiment, whereas broilers in other treatment groups received a standard diet plus FNSP at 0.75% (T2), standard diet plus FNSP at 1.75% (T3), standard diet plus CNBP at 0.75% (T4), and standard diet plus CNBP at 1.75% (T5). Commercial starter feed was purchased from Alema Koudijs Feed PLC, and finisher feed was from Mulie Animal Feed Processing Factory, located in Bishoftu, Ethiopia. Chicks were fed diets at their respective ages (starter feed from day 1–28 days, and finisher feed from day 29–49 days). The daily required amount of feed was weighed every morning and offered to each treatment twice at 07:00 a.m. and 05:00 p.m. Clean water was offered *ad libitum* during the experimental period*.* Daily amount of feed offered was adjusted based on the chicks’ weight measured every week throughout the experiment.

### 2.4. Animal Management

Before the arrival of the chicks, the broiler shed, its premises, and the required equipment were thoroughly cleaned and disinfected. The pens were cleaned using tap water mixed with detergent and left to air dry for 3 days. After thorough drying, the entire poultry house was disinfected with 50% hydrogen peroxide (H_2_O_2_), diluted at one liter for every 16 liters of water, using a spray application method. The floor of each pen was covered with disinfected *Teff* (*Eragrostis Teff*) straw as a bedding material to a depth of 10 cm. The broilers were managed in a partitioned pen each with a floor area of 1.2 × 1.8 m on a deep litter floor system. During the first 15 days of rearing, one plastic tray feeding per pen was used and then they were replaced by a proper type feed trough at the start of the third week. The chicks were brooded using 200‐W bulbs suspended with gradual height adjustment as heat and light sources for each pen. The heat lamps were adjusted at 50 cm off the floor for the first week for each pen and then reduced by 8 cm each week during the brooding period. The initial temperature of 32°C was maintained in the first week and thereafter reduced by 3°C each week until reaching the room temperature of 20°C by the end of the fourth week. The light was continuous for the first 48 h following chick placement, and then, it was provided 23 h per day for the rest of the experimental period. Chickens were vaccinated against major diseases on a routine basis in line with the local commercial broilers’ vaccination programs of the Alema farms. All chickens were vaccinated against Vitabron L plus IBD using spray technique and Transmune and Vectormune ND via subcutaneous (SC) at hatchery. Newcastle thermostable (NCTH) vaccines were administered at 10 and 20 days of age through drinking water. Likewise, the experimental chicks were vaccinated against infectious bursal disease (IBD) at 14 and 21 days of age through drinking.

### 2.5. Chemical Analysis of Feed

For proximate composition determination, feed offered and refused per treatment during starter and finisher phases was sampled and bulked. Samples of feeds offered and refused were subsampled for each phase. The representative samples were ground to pass through a 1‐mm screen for chemical composition analysis. Feed composition analysis was conducted at the Animal Nutrition Laboratory of Haramaya University, Ethiopia.

All feed samples were analyzed for dry matter (DM), crude fiber (CF), total ash, ether extract (EE), and Kjeldahl nitrogen (N) according to AOAC [23]. The CP content was calculated as N × 6.25. Calcium and phosphorus contents were determined using the atomic absorption spectrophotometer method and photometric method, respectively, according to AOAC [23]. The metabolizable energy (ME) content of the experimental diets was calculated by the indirect method using the equation proposed by Wiseman [24] as ME (kcal/kg DM) = 3951 + 54.4 EE − 88.7 CF − 40.8 ash, where: ME = metabolizable energy, EE = ether extract, and CF = crude fiber.

### 2.6. Feed Intake

The daily amount of feed consumed by the respective treatment was determined as the difference between the amount of daily feed offered and the amount refused. Impurities and foreign materials that might have been mixed with the feed in the trough were manually separated before weighing of the refusal feed. The average feed intake per bird was determined as the difference between the feed offered and refused divided by the number of broilers:

(1) average feed intake/bird g=feed offered g−feed left over gnumber of birds per replicate.

### 2.7. BW Gain

BW was measured for each replication group at the beginning of the experiment (at 5 days old) and then weekly. Broilers were weighed in a group per pen, and the pen average was calculated. BW changes were calculated as the difference between the final and initial BW (IBW). Average daily weight gain (ADWG) was calculated as the ratio of BW change to the number of experimental days. The FCR (feed consumed to produce a unit of gain) was calculated from the ratio of the average amount of feed consumption and average weight gain:

(2) feed conversion ratio FCR=amount of feed consumed ggain in body weight g.

### 2.8. Carcass Characteristics

At the end of the experiment, three broilers per treatment were selected for measurements of carcass traits. All broilers were starved for about 12 h (overnight fasting); however, drinking water was provided *ad libitum*. To measure carcass production potential, one broiler chicken per replicate with a BW exceeding the average BW of that replicate was selected for slaughter after overnight fasting. The broilers selected for slaughter were separated and leg tagged for identification and kept in a separate pen until slaughtering. The broilers picked for carcass measurement were weighed individually and then slaughtered by severing the throat through the jugular vein using a sharp knife and allowed to bleed completely. Following complete bleeding, the carcass was defeathered by hand plucking after scalding in hot water for about 2 min and their heads and shanks were removed. The dressed weight was measured, and the dressing percentage was computed as the proportion of the carcass weight to slaughter weight multiplied by one hundred. The abdominal cavity of the dressed carcass was opened, and the viscera were removed to determine the eviscerated carcass weight. The eviscerated carcass weight was measured after removal of blood, feathers, shanks, head, kidney, lungs, pancreas, crop, proventriculus, small intestine, large intestine, caeca, and urogenital tracts. From the eviscerated carcass, the primal cuts of the carcass (drumstick, thigh, breast, back, neck, and wings) were separated, weighed, and expressed relative to the slaughter BW:

(3) eviscerated percetage %= eviscerated weight glive weight g×100.

The giblets (heart, liver, and gizzard) were trimmed from the viscera, weighed, and determined as a proportion of slaughter weight. The different standards for the measurements of carcass traits were computed according to the methods employed by FAO [25].

### 2.9. Statistical Analysis

The data were analyzed following one‐way ANOVA using GLM procedures of statistical analysis systems, SAS (2004 Ver 9.1) with a model containing treatment as the main effect and pen as an experimental unit. The assumptions for all variables were verified using the Shapiro–Wilk test for normality and Bartlett’s test for homogeneity of variances. Duncan’s multiple comparison test was used to compare the differences among the treatment means, and *p* < 0.05 was considered significant. The statistical model used in the study was

(4) Yij=μ+Ti+Eij,

where *Y*_*i* *j*_ is the value of the measured variable, *μ* is the overall mean, *T*_*i*_ is the effect of the treatment (*i* = five treatments), and *E*_*i* *j*_ is the residual error.

## 3. Results

### 3.1. Growth Performance

The effects of adding FNSP and CNBP at different levels to broiler diets on the feed intake and BW gains are summarized in​ Table 1. Dietary treatments did not show any significant effects on the feed intake; however, an improved feed intake was found in the starter phase (*p* = 0.066) and entire experiment period (*p* = 0.075) in broilers fed a diet containing 1.75% FNSP followed by 0.75% CNP when compared to other treatment groups including the control. In this study, broiler chickens fed diets incorporated with 0.75% FNSP had a significantly (*p* = 0.006) higher final body weight gain (FBWG) of 1269 g/bird than broilers in the control group (1208 g/bird) during the starter phase.

**TABLE 1:** Effects of dietary fennel and cinnamon on feed intake and growth performance of Cobb 500 broilers.

Parameters	Treatments						
	Control	0.75% FNSP	1.75% FNSP	0.75% CNBP	1.75% CNBP	SEM	*p* value
IBW (g/bird) at day 5	124.74	120.62	116.97	120.65	123.89	1.14	0.201
Starter phase (6–28 days)							
FI (g/bird)	1802.61	1800.80	1821.20	1809.76	1789.82	3.72	0.066
FBWG (g/bird)	1208.40^b^	1268.52^a^	1197.85^b^	1170.25^b^	1213.56^b^	10.03	0.006
TBWG (g/bird)	1083.66^b^	1147.90^a^	1080.88^b^	1049.60^b^	1089.67^b^	9.84	0.004
ADWG (g/bird)	47.12^b^	49.91^a^	46.99^b^	45.64^b^	47.38^b^	0.43	0.004
FCR	1.66^ab^	1.57^c^	1.69^ab^	1.72^a^	1.64^b^	0.02	0.006
Finisher phase (29–49 days)							
IBW (g/bird)	1208.40^b^	1268.52^a^	1197.85^b^	1170.25^b^	1213.56^b^	10.03	0.006
FI (g/bird)	2715.30	2715.47	2716.71	2717.05	2715.33	0.36	0.395
FBWG (g/bird)	2778.10^b^	2840.70^b^	3016.80^ab^	2818.20^b^	3243.00^a^	58.88	0.036
TBWG (g/bird)	1569.70^b^	1572.18^b^	1818.95^ab^	1647.95^b^	2029.44^a^	60.60	0.039
ADWG (g/bird)	74.75^b^	74.87^b^	86.62^ab^	78.47^b^	96.64^a^	2.89	0.039
FCR	1.73^a^	1.73^a^	1.49^ab^	1.65^a^	1.34^b^	0.05	0.038
Overall (6–49 days)							
FI (g/bird)	4517.92	4516.27	4537.90	4526.81	4505.15	3.98	0.075
FBW (g/bird)	2778.10^b^	2840.70^b^	3016.80^ab^	2818.20^b^	3243.00^a^	58.88	0.036
TBWG (g/bird)	2653.36^b^	2720.08^b^	2899.83^ab^	2697.55^b^	3119.11^a^	58.87	0.036
ADWG (g/bird)	60.30^b^	61.82^b^	65.91^ab^	61.31^b^	70.88^a^	1.20	0.036
FCR	1.70^a^	1.66^a^	1.57^ab^	1.68^a^	1.44^b^	0.03	0.033

*Note:* ^abc^Means with different superscript letters within the same row differ significantly (*p* < 0.05); CNBP: cinnamon bark powder; FNSP: fennel seed powder; T1: control group; T2: standard feed plus 0.75% FNSP; T3: standard feed plus 1.75% FNSP; T4: standard feed plus 0.75% CNBP; T5: standard feed plus 1.75% CNBP; FBW: final body weight; TBWG: total body weight gain; FI: feed intake; ADWG: average daily weight gain.

Abbreviations: FCR, feed conversion ratio; IBW, initial body weight; SEM, standard error of the mean; SF, standard feed.

During the finisher phase, significantly (*p* = 0.036) higher FBWG was recorded in broiler chickens that fed diets with CNBP at 1.75%, compared to those fed a basal diet without any additives. Additionally, broilers fed a diet containing 0.75% FNSP exhibited significantly (*p* = 0.004) higher total body weight gain (TBWG) and ADWG than other treatments at the starter phase (Table 1). At the finisher phase, broilers fed diets with CNBP at 1.75% had significantly (*p* = 0.039) higher TBWG and ADWG, while broilers in the control group had the lowest TBWG and ADWG. Similarly, significantly (*p* = 0.036) higher TBWG and ADWG were observed with the addition of 1.75% CNBP in the overall experiment period. As indicated in Table 1, the inclusion of cinnamon and fennel as natural feed additives in broilers’ diet resulted in a significant difference between treatments in terms of FCR. At the starter phase, broilers fed 0.75% FNSP had significantly (*p* = 0.006) lower FCR compared with those in the control group, while in the finisher phase, significantly (*p* = 0.038) lower FCR was noted in broilers fed diets containing CNBP at 1.75%. Moreover, significantly lower (*p* = 0.033) FCR was observed in broilers fed 1.75% CNBP than in broilers in the control group during the entire experiment period.

### 3.2. Carcass Characteristics

The effects of fennel and cinnamon powder on the slaughter, dressed, and eviscerated weight and primal carcass traits of broiler chickens are presented in Tables 2 and 3, respectively.

**TABLE 2:** Effects of dietary fennel and cinnamon on slaughter, dressed, and eviscerated weight of Cobb 500 broilers.

Parameters	Treatments						
	Control	0.75% FNSP	1.75% FNSP	0.75% CNBP	1.75% CNBP	SEM	*p* value
Live weight (g)	3227	3127	3310	3193	3513	48.95	0.083
Dressed weight (g)	2947^b^	2883^b^	3060^ab^	2968^b^	3323^a^	53.10	0.039
Dressing percentage (%)	91.32	92.20	92.45	92.95	94.59	0.48	0.312
Eviscerated weight (g)	2333^c^	2380^bc^	2513^ab^	2507^abc^	2677^a^	37.85	0.008
Eviscerated percentage (%)	72.30	76.11	75.92	78.52	76.20	0.97	0.466

*Note:* ^abc^Means with different superscript letters within the same row differ significantly (*p* < 0.05); CNBP: cinnamon bark powder; FNSP: fennel seed powder; T1: control group; T2: standard feed plus 0.75% FNSP; T3: standard feed plus 1.75% FNSP; T4: standard feed plus 0.75% CNBP; T5: standard feed plus 1.75% CNBP.

Abbreviations: SEM, standard error of the mean; SF, standard feed.

**TABLE 3:** Effects of dietary fennel and cinnamon on the primal carcass traits in Cobb 500 broiler chickens.

Parameters	Treatments						
	Control	0.75% FNSP	1.75% FNSP	0.75% CNBP	1.75% CNBP	SEM	*p* value
Breast weight (g)	788.00^b^	790.00^b^	935.67^a^	942.67^a^	931.33^a^	21.14	0.001
Breast (%)	24.44^c^	25.29^c^	28.28^ab^	29.59^a^	26.53^bc^	0.61	0.012
Back weight (g)	336.00	322.33	327.67	324.00	377.33	8.33	0.202
Back (%)	10.46	10.33	9.90	10.17	10.75	0.25	0.898
Drumstick weight (g)	310.67	313.67	315.33	305.67	324.33	2.18	0.058
Drumsticks (%)	9.67	10.01	9.53	9.59	9.24	0.18	0.611
Thigh weight (g)	383.67^b^	393.33^b^	397.33^b^	430.00^a^	416.33^a^	4.87	0.001
Thigh (%)	11.94^b^	12.58^ab^	12.01^b^	13.49^a^	11.86^b^	0.21	0.047
Whole leg weight (g)	694.33^b^	707.00^b^	712.67^b^	735.67^a^	740.67^a^	5.15	0.001
Whole leg weight (%)	21.61	22.62	21.54	23.09	21.10	0.32	0.273
Neck weight (g)	116.67^bc^	130.00^ab^	109.67^c^	113.67^c^	132.33^a^	2.96	0.017
Neck (%)	3.63^b^	4.16^a^	3.31^b^	3.56^b^	3.77^ab^	0.09	0.034
Wing weight (g)	238.00	242.00	231.67	230.67	238.33	2.49	0.471
Wings (%)	7.42	7.74	7.00	7.24	6.79	0.15	0.271

*Note:* ^abc^Means with different superscript letters within the same row differ significantly (*p* < 0.05); CNBP: cinnamon bark powder; FNSP: fennel seed powder; T1: control group; T2: standard feed plus 0.75% FNSP; T3: standard feed plus 1.75% FNSP; T4: standard feed plus 0.75% CNBP; T5: standard feed plus 1.75% CNBP.

Abbreviations: SEM, standard error of the mean; SF, standard feed.

In this study, slaughter weight along with dressing and eviscerated percentage did not show any significant difference across treatments (*p* > 0.05). However, broilers in the treatment group of 1.75% CNBP exhibited a significantly (*p* = 0.039) higher dressed weight in comparison with the control and other treatment groups. Additionally, increased dressed weight (3060 g) was observed in the 1.75% FNSP group as compared to the control (2947 g), though not significant (Table 2). Similarly, broilers fed 1.75% CNBP had a significantly (*p* = 0.008) higher eviscerated weight compared to the control and other treatment groups. The CNBP at a higher level of inclusion (1.75%) used in this study resulted in a significant improvement in terms of dressed and eviscerated carcass weights compared to the control, while 1.75% FNSP showed significance compared with the control group in the eviscerated weight.

#### 3.2.1. Primal Cuts

Inclusion of CNBP and FNSP in broiler diets at different levels (0.75% and 1.75%) significantly affected (*p* < 0.05) the components of primal carcass cuts, including breast weight, breast percentage, thigh weight, thigh percentage, whole leg weight, neck weight, and neck percentage (Table 3). On the other hand, the addition of fennel and cinnamon at all levels used in this study did not affect the weight and percentage of back, drumsticks, and wings. Significantly (*p* = 0.001) higher breast weight was observed with the addition of 1.75% CNBP, 1.75% FNSP, and 0.75% CNBP compared to the control group. Moreover, recorded thigh weight in broilers fed 1.75% CNBP and 0.75% CNBP was significantly (*p* = 0.001) higher than in those fed the control diet. Significantly (*p* = 0.001) higher whole leg weight (thigh plus drumstick) was observed by the inclusion of CNBP at 1.75% and 0.75% levels. Furthermore, broilers fed diets with 1.75% CNBP showed a significantly (*p* = 0.017) higher neck weight compared to broilers fed a standard diet with no feed additives.

#### 3.2.2. Giblet Yield

As summarized in Table 4, inclusion of CNBP at 1.75% in the diet of broiler chickens had a significant effect on the primal carcass cuts and edible carcass weights compared to the control and other treatments, except for the 0.75% CNBP group. On the other hand, broilers fed diets with 0.75% CNBP showed significantly higher gizzard weight and its relative weight over all other treatment groups including the control. Similarly, diets with the addition of 0.75% CNBP exhibited significantly higher giblet relative weight in comparison with the control and other treatments. Broilers fed a diet containing 1.75% CNBP had significantly (*p* = 0.001) higher primal carcass weight of 2420 g, followed by 2347 g in those fed 0.75% CNBP compared with the control group (2173 g). Additionally, broilers fed CNBP at a level of 1.75% demonstrated higher edible carcass (carcass plus giblet) production than those in the control group (*p* = 0.001). Moreover, there was a significant (*p* = 0.014) rise in giblet percentage in groups fed 0.75% CNBP as compared with the control group. At the end of the experiment, the average relative gizzard weight for each treatment was 1.19% in the control, 1.34% in the 0.75% FNSP, 1.25% in the 1.75% FNSP, 1.77% in the 0.75% CNBP, and 1.10% in the 1.75% CNBP. The 0.75% CNBP group showed significantly higher (*p* = 0.006) gizzard weight and relative weight (*p* = 0.002) compared to the control group, whereas the heart and liver mean value weights were not significantly (*p* > 0.05) affected by adding FNSP and CNBP to the broiler diet at 0.75% and1.75% inclusion levels.

**TABLE 4:** Effects of dietary fennel and cinnamon on carcass and giblet yield of Cobb 500 broiler chickens.

Parameters	Treatments						
	Control	0.75% FNSP	1.75% FNSP	0.75% CNBP	1.75% CNBP	SEM	*p* value
Primal carcass weight (g)	2173.00^c^	2191.33^c^	2317.33^b^	2346.67^ab^	2420.00^a^	27.39	0.001
Primal carcass (%)	67.57	70.13	70.03	73.65	68.93	0.96	0.386
Heart weight (g)	19.67	22.00	20.00	22.00	20.00	0.61	0.627
Heart (%)	0.61	0.71	0.60	0.69	0.57	0.02	0.354
Liver weight (g)	58.00	60.67	65.33	62.33	64.67	1.93	0.805
Liver (%)	1.80	1.94	1.97	1.95	1.83	0.05	0.831
Gizzard weight (g)	38.33^b^	42.00^b^	41.33^b^	56.33^a^	38.00^b^	2.11	0.006
Gizzard (%)	1.19^b^	1.34^b^	1.25^b^	1.77^a^	1.10^b^	0.07	0.002
Giblet weight (g)	116.00	124.67	126.67	140.67	122.67	3.01	0.103
Giblet (%)	3.60^b^	3.99^ab^	3.82^b^	4.41^a^	3.49^b^	0.10	0.014
Edible carcass (g)	2289.00^c^	2316.00^c^	2444.00^b^	2487.33^ab^	2542.67^a^	28.49	0.001
Edible carcass (%)	71.18	74.12	73.86	78.07	72.42	1.02	0.280

*Note:* ^abc^Means with different superscript letters within the same row differ significantly (*p* < 0.05); CNBP: cinnamon bark powder; FNSP: fennel seed powder; T1: control group; T2: standard feed plus 0.75% FNSP; T3: standard feed plus 1.75% FNSP; T4: standard feed plus 0.75% CNBP; T5: standard feed plus 1.75% CNBP.

Abbreviations: SEM, standard error of the mean; SF, standard feed.

## 4. Discussion

### 4.1. Growth Performance of Broiler Chickens in Response to Dietary Inclusion of Cinnamon and Fennel

In this study, the inclusion of cinnamon and fennel in the diets of broiler chickens resulted in a significant improvement in terms of FBWG, ADWG, and FCR. On the other hand, the diets with the addition of CNBP and FNSP had no significant effect on the feed intake; however, an improved feed intake in the starter phase (*p* = 0.066) and overall feeding period (*p* = 0.075) was observed, though not significant. This suggests that dietary FNSP and CNBP did not adversely affect the acceptability of the diet, probably due to the better tolerance of broiler chickens to bitter taste and warm and intense spicy smell of cinnamon and fennel. The results are in line with that of Nassar et al. [8], who found that the addition of FNSP (at 10, 20, and 30 g/kg) to the broiler diet had no significant impact on the feed consumption. Similarly, Al‐Sagan et al. [6] reported that inclusion of 1.6% and 3.2% fennel seed additives to broiler diet under normal temperatures resulted in insignificant differences in the feed intake except under heat stress conditions. Moreover, the present results are consistent with those reported by Chowlu et al. [26], who found that the overall FI in broilers provided with cinnamon powder at 2.5, 5.0, and 7.5 g/kg diet for a period of 42 days did not vary significantly as compared to the control group. Conversely, there are some study findings that contradict the present result regarding the effects of dietary inclusion of cinnamon on the feed intake of broilers. For instance, Saki et al. [27] reported that providing broilers with diet containing 0.5% FNSP increased feed intake, while Nath et al. [28] observed a significant decrease in the feed intake during the entire experiment (0–42 days) period in broilers fed a basal diet plus 100 mg/kg cinnamon oil plus 2 g/kg neem leaf powder compared to the control.

With regard to growth performance, broilers fed diets with 1.75% CNBP demonstrated significantly (*p* = 0.036) higher BW and ADWG than those fed the control diet in the overall feeding trial. Likewise, significantly improved FCR (*p* = 0.033) was observed in broilers fed a diet containing 1.75% CNBP followed by 1.75% FNSP, indicating that the 1.75% CNBP group required less feed to gain more BW than all treatments. These improvements in growth performance might be attributed to the presence of bioactive compounds in cinnamon and fennel, which could have beneficial effects for stimulating and activating the digestive system, which facilitate digestion and nutrient utilization in broilers [16, 29], resulting in improved broiler chickens’ growth rates and FCR. In the present study, broilers fed dietary cinnamon showed higher growth performances in comparison with fennel‐fed groups. These beneficial functions could be attributed to the existence of more effective main bioactive constituents, cinnamaldehyde and other ingredients such as eugenol in cinnamon bark, which help to increase the activity of digestive enzymes, facilitating digestion and nutrient utilization, enhancing the overall growth performance of broiler chickens [10, 14]. Surai [30] and Bakeer et al. [15] elucidated that improvement in growth performance in animals could be associated with the growth‐enhancing effect of phenolic compounds that lead to increased digestion and nutrient absorption for tissue synthesis.

In consistent with the present results, several other studies have also reported positive effects of dietary fennel and cinnamon powder on growth performance, specifically in terms of BWG, ADWG, and FCR in broilers. Nassar et al. [8] reported a significant linear rise in the FBWG and BWG when FNSP was incorporated at 10, 20, and 30 g/kg into the diet of broilers. Saleh et al. [31] also found that broilers fed 750 g of FNSP per 50 kg of diet demonstrated significantly higher BW in comparison with the control. In addition, Abdel‐Raheem et al. [7] reported that broilers fed FNSP at 1.2% had significantly higher BWG at 49 days of age compared to the control. The study conducted by Shirzadegan [32] found that the addition of cinnamon powder, especially at 0.50%, resulted in the best FCR and increased FBWG in broilers. Moreover, Khafaji [33] found that broilers fed a diet with 0.5 and 1 g/kg of CNBP exhibited significantly increased BWG compared to the control at the end of the experiment. Furthermore, Saki et al. [27] reported a significant improvement in terms of FCR in broilers fed a basal diet with 0.5% FNSP in comparison with broilers in the control.

On the other hand, the inclusion of FNSP to Cobb 500 broilers at 0.15%–0.45% levels in the study conducted by Liu et al. [16] did not affect BWG, ADWG, and FCR, which contradicts the present results. Additionally, Chowlu et al. [26] observed no difference in the performance in terms of weight gain and feed conversion efficiency in broilers received a diet containing CNBP at 2.5–7.5 g/kg inclusion levels for 42 days in comparison with the control group. Moreover, Bezahegn et al. [34] reported that the addition of fennel and cinnamon powders to the diet at inclusion levels of 0.75% and 1.75% had no significant effect on the feed intake, final weight gain, and BW change; however, improved production performance and quality characteristics of egg were observed in Lohmann brown laying hens. Based on the reviewed literature, the varied investigations among different studies may be associated with type and quality of basic feed, the inclusion level of the test diet, environment, and length of feeding trial [35, 36].

### 4.2. Effects of Dietary Inclusion of Cinnamon and Fennel on Carcass Traits in Broilers

The results revealed that broiler chickens fed a diet containing 1.75% CNBP followed by 1.75% FNSP had significantly higher dressed and eviscerated weight than the control. Similarly, broiler chickens fed diets containing 1.75% CNBP showed higher primal cuts including thigh and whole leg weight compared with the control and other treatment groups, while breast weight was positively influenced by the 1.75% FNSP and CNBP at both levels used in this study. Likewise, neck weight was significantly higher in the broilers fed diets with 1.75% CNBP when compared to the control.

These improvements in terms of dressed, eviscerated, and primal carcass yields may be due to the growth‐ and development‐promoting effects of biologically active substances found in fennel and cinnamon, resulting in heavier slaughter BW likely associated with improved carcass production. The results agreed with those of Khan et al. [37], who reported significantly higher BWG, FCR, dressing percentage, and breast and thigh weights in broilers receiving CNBP at 1% inclusion level. Nassar et al. [8] also found that broilers fed diets with FNSP at 20 g/kg of diet produce increased breast yield in comparison with other treatment groups.

Concerning giblets, the average relative gizzard weight for CNBP at the 0.75% inclusion level was significantly higher, while no significant differences among treatments were observed in the weight and relative weight of the liver and heart. The absence of significant differences indicates that neither the liver nor the heart was adversely affected by the dietary cinnamon and fennel additives at the inclusion levels of 0.75% and 1.75%. Significantly higher gizzard weight observed in broilers fed diets with the CNBP may be linked with the higher fiber levels found in the CNBP‐containing diet (Table 5) when compared to the control diet, possibly leading to an increased coarseness of the diet, which may have contributed to the development of the gizzard. Zaefarian et al. [38] stated that increased coarseness of the diet will influence the gizzard development, since it stimulates gastric functions, resulting in better grinding of feed. The results of this study were supported by those of Al‐Kassie [39], who reported that broilers fed diets with cinnamon extract at a concentration of 200 mg/kg had a significantly higher relative gizzard weight in comparison with the control group. Overall, this study revealed that inclusion of dietary CNBP and FNSP, particularly CNBP at a level of 1.75%, did seem to noticeably improve BW gain, FCR, and carcass traits in broilers, indicating better meat production performance. However, due to the small number of experimental samples used for carcass evaluation, the effects of dietary CNBP and FNSP on carcass traits in broiler chickens need to be studied further.

**TABLE 5:** Proximate composition of treatment diets fed to Cobb 500 broiler chickens.

Proximate composition (%)	DM	CP	EE	CF	Ash	Ca	P	ME (kcal/kg)
*Starter phase diet (1–28 days)*								
T1 (standard feed, SF)	89.89	21.35	4.18	6.90	11.00	1.34	0.75	3118
T2 (SF+ 0.75% FNSP)	90.17	21.51	4.12	7.31	9.89	1.27	0.72	3123
T3 (SF+ 1.75% FNSP)	90.36	21.54	4.11	7.13	9.96	1.26	0.69	3136
T4 (SF+ 0.75% CNBP)	89.83	21.49	3.99	7.12	9.65	1.06	0.65	3143
T5 (SF+ 1.75% CNBP)	89.64	21.54	3.96	7.10	9.66	0.96	0.61	3143

*Finisher phase diet (29–49 days)*								
T1 (standard feed, SF)	90.75	18.75	5.43	5.81	9.94	1.09	0.68	3326
T2 (SF+ 0.75% FNSP)	91.49	18.82	5.39	6.28	8.72	1.06	0.65	3331
T3 (SF+ 1.75% FNSP)	91.52	18.89	5.36	6.26	8.54	1.02	0.63	3339
T4 (SF+ 0.75% CNBP)	91.15	18.78	5.29	6.21	8.37	0.92	0.58	3347
T5 (SF+ 1.75% CNBP)	91.19	18.82	5.22	6.11	8.32	0.91	0.54	3354

*Note:* CNBP= cinnamon bark powder; FNSP= fennel seed powder; Ca=calcium; P = phosphorus; T1 = control group; T2 = standard feed plus 0.75% FNSP; T3 = standard feed plus 1.75% FNSP; T4 = standard feed plus 0.75% CNBP; T5 = standard feed plus 1.75% CNBP.

Abbreviations: CF, crude fiber; CP, crude protein; DM , dry matter; EE, ether extract; ME, metabolizable energy (kcal/kg); SF, standard feed.

## 5. Conclusion

The results of this study showed that the addition of CNBP at 1.75% in the broiler chickens’ diet significantly improved performance in terms of BW gain, ADWG, and FCR followed by 1.75% FNSP. Additionally, dietary CNBP at an inclusion level of 1.75% exhibited superior performance in some carcass traits, including dressed and eviscerated weight, and weight of carcass cut components (breast, thigh, whole leg meat, and neck) in broilers. On the whole, while the addition of both fennel and cinnamon powders in the diet of broiler chickens tended to be beneficial natural feed additives, dietary cinnamon powder mainly at a level of 1.75% resulted in significant improvements in overall growth performance and carcass traits.

## Author Contributions

Abebe Bezahegn conceptualized the research idea, collected the data, conducted formal data analysis and data curation, and drafted the original manuscript. Berhan Tamir and Gezahegne Mamo were involved in supervision of the study, conceptualization, and validation and reviewed and edited the manuscript.

## Funding

The research was financially supported by the Ministry of Education through the College of Veterinary Medicine and Agriculture of Addis Ababa University.

## Disclosure

All authors have read and approved the final version of the manuscript.

## Ethics Statement

All experimental procedures related to animal handling and their management procedures followed the animals care guidelines and protocols approved by the Institutional Animal Ethics Committee of the College of Veterinary Medicine and Agriculture, Addis Ababa University (CVMA‐AAU), Ethiopia (Reference No. VM/ERC/01/13/12/2020).

## Conflicts of Interest

The authors declare no conflicts of interest.

## Data Availability

The data used during the present study are available from the corresponding author upon reasonable request.
